# Using the Khorana risk score to predict venous thromboembolism and overall survival in a cohort of Hispanic patients with solid malignancies

**DOI:** 10.3332/ecancer.2022.1470

**Published:** 2022-11-10

**Authors:** Allan Ramos-Esquivel, Ana Marenco-Flores, Gabriel Hernández-Romero, Carlos Umaña-Mora, Ana Céspedes-Calvo, Raquel Mora-Hidalgo

**Affiliations:** 1 Medical Oncology Service, Hospital San Juan de Dios, Caja Costarricense de Seguro Social, 1001, Costa Rica; 2 Department of Pharmacology, School of Medicine, University of Costa Rica, Sede Rodrigo Facio, 2082 San Pedro, San José, Costa Rica; 3 Department of Medicine, NYC, New York, Jacobi Medical Center/Albert Einstein College of Medicine, New York, NY 10461, USA

**Keywords:** Hispanic, mortality, neoplasms, prognosis, venous thromboembolism

## Abstract

**Background:**

The Khorana risk score (KRS) for prognosis of venous thromboembolism (VTE) has been rarely explored in Hispanic populations.

**Objective:**

To determine the value of the KRS for prediction of VTE and overall survival (OS) among Hispanic individuals with cancer.

**Methods:**

We retrospectively evaluated all outpatients with newly diagnosed solid tumours receiving systemic chemotherapy in Hospital San Juan Dios, San José, Costa Rica, from January to December 2021. The 6-month cumulative VTE incidence according to the KRS categories was estimated using the Fine & Gray competing risk model. A Kaplan–Meier analysis was used to compare OS among KRS categories. The Cox regression analysis was performed to calculate the hazard ratio (HR) and its corresponding 95% confidence interval (CI). The receiver operating characteristic (ROC) analysis was performed to identify the optimal cutoff value to predict VTE during follow-up.

**Results:**

A total of 708 patients were included in the analysis. After a median follow-up of 8.13 months, the cumulative incidence of VTE at 6 months was 1.56% (95% CI: 0.83%–6.82%), 4.83% (95% CI: 2.81%–7.66%) and 8.84% (95% CI: 4.30%–15.42%) for low-, intermediate- and high-risk Khorana score categories, respectively (Gray’s *p* value: 0.0178). The optimal cutoff for the KRS to predict VTE was 2 (area under the ROC curve: 0.65; 95% CI: 0.55–0.756). The KRS was independently associated with overall mortality (HR: 1.83; 95% CI: 1.46–2.29; *p* < 0.001, for the comparison of ‘high-risk’ and ‘low-risk’ KRS).

**Conclusions:**

The KRS is a valid tool to predict VTE and mortality in a cohort of Hispanic outpatients with newly diagnosed solid tumours.

## What is known on this topic?

- The Khorana risk score (KRS) is a recommended tool to assess the risk of venous thromboembolism (VTE) in patients with cancer.
- Ethnic and clinical variables affect the risk of VTE events in patients with cancer and it is largely unknown if the KRS predicts the probability of VTE in patients of Hispanic origin.

## What does this paper add?

- In this retrospective cohort study, we demonstrated the validity of the KRS to identify outpatients with solid tumours at risk for VTE during chemotherapy.
- The KRS was associated with VTE events and poor survival in the studied population. Therefore, we recommend its use to identify patients in which prophylactic anticoagulation should be discussed.

## Introduction

Thromboembolic disease is an entity comprised by the thrombotic event of venous thromboembolism (VTE). This includes pulmonary embolism and deep venous thrombosis [1]. VTE is one of the main causes of morbidity, and the second most frequent cause of death in cancer patients [2–4]. This population has a four times higher risk of thrombosis compared to the general population [2]. Current evidence shows that 1%–15% of patient with cancer will develop VTE during the course of their disease [3–5].

The risk of thromboembolic disease must be evaluated in the beginning and periodically in patients with cancer, particularly in those receiving systemic therapy. Accordingly, the need of thromboprophylaxis is evaluated by means of validated score systems [6, 7]. One of the main contemporary risk prediction scores is the Khorana risk score (KRS), being the most validated, and endorsed by the guidelines of the American Society of Clinical Oncology and the American Society of Hematology, for risk stratification of thromboembolism in ambulatory cancer patients [6–9]. This score was created in 2008 by Khorana *et al* [8] as a predictive model for VTE associated to chemotherapy in ambulatory patients. It uses two clinical variables (site of tumour and body mass index) and three laboratory measurements (platelets, haemoglobin and leucocyte count) [8]. The calculation stratifies individuals among three risk categories. This targeted approach allows thromboprophylaxis to be prescribed in patients with higher risk of VTE while avoiding the risk of haemorrhage in those with low scores [6, 7]. Although the KRS has been validated in several populations [5, 9–11], we found scarce data regarding the prognostic value of this tool among Hispanic patients from Latin American countries. Indeed, previous studies have described that Hispanic patients have a higher incidence of VTE in comparison to non-Hispanic Whites and Asian individuals, but a lower frequency of VTE in comparison to African American patients [12–14]. Moreover, recent studies have associated the KRS as a prognostic variable for overall survival (OS) in cancer patients, but most of these analyses have been carried out in different populations other than Hispanics [3]. Therefore, this study aims to determine the prognostic value of the KRS to predict VTEs and mortality in a cohort of Hispanic outpatients with solid tumours.

## Methods

A retrospective closed cohort study was carried out at the outpatient Oncology Clinic of San Juan de Dios Hospital (Caja Costarricense de Seguro Social), San José, Costa Rica. We included all Hispanic ambulatory patients with newly diagnosed solid tumours who received intravenous chemotherapy from 1 January to 1 December 2021. For the purpose of this study, we defined Hispanic as any person of Cuban, Mexican, Puerto Rican, South or Central American or other Spanish culture or origin, regardless of race, as determined by the treating oncologist at first consultation [15].

We excluded patients on immunotherapy, targeted therapy and anti-hormonal treatment. Other exclusion criteria were insufficient documentation in electronic medical records, past medical history of VTE and current use of prophylactic or therapeutic anticoagulation. Patients were followed-up for at least 6 months, from the initiation of chemotherapy until death (as recorded in the Costa Rican National Registry) or until the date of censoring (31 May 2022).

Medical records were examined to calculate the KRS at the beginning of therapy and to determine the occurrence of VTE and death during follow-up. Briefly, the KRS consists of five clinical items: tumour site (stomach and pancreatic cancers are classified as ‘very-high-risk’ and assign 2 points to the score; lung, lymphoma, gynaecological, bladder or testicular cancer are classified as ‘high-risk’ and score 1 point), prechemotherapy platelet count of more than 350,000/µL, haemoglobin concentration of less than 10 g/dL and/or use of erythropoiesis-stimulating agents, white blood cells more than 11,000/µL and body mass index of more than 35 Kg/m^2^. Each of these variables assigns 1 point to the KRS. Patients with an overall score of 0 points are classified as ‘low risk’, 1 to 2 points as ‘intermediate-risk’ and ≥3 points as ‘high risk’ for VTE [8].

The primary outcome was the diagnosis of VTE, defined as the development of symptomatic proximal or distal deep-vein thrombosis, pulmonary embolism or both at any time between the initiation of systemic treatment and the end of follow-up. All cases of VTE were objectively confirmed by assessment of both clinical records and radiological reports. Only one VTE event per patient was counted, regardless of whether they experienced multiple episodes. VTE events were retrospectively extracted from electronic medical records by one of the co-authors (AM) who was unaware of the clinical prediction score calculation. A secondary outcome was to determine the prognostic association between the KRS and overall mortality in the overall population.

### Statistical analysis

Categorical variables are expressed as frequencies and percentages, while continuous variables are presented as means and standard deviations, or medians and interquartile ranges, as appropriate. The sixth-month cumulative VTE incidence for the overall population and according to the KRS categories was estimated using the Fine & Gray competing risk model [16], which considered non–VTE-related death as competing risk. The Gray’s test was used to compare the cumulative incidence functions across groups of risk.

The area under the receiver operating characteristic (ROC) curve was calculated to determine the overall performance of the KRS in the prediction of VTE. The optimal cutoff of the KRS for predicting VTE, as well as its sensitivity and specificity at each point, was determined via ROC analysis.

The association between KRS categories and OS was examined using a Kaplan–Meier survival curve and a Cox proportional-hazard regression model, using Eastern Cooperative Oncology Group (ECOG) and age as covariates. The log-rank test was used to compare the distributions of OS among KRS groups.

The statistical analyses were done with SAS 9.4 (SAS Institute Inc., Cary, North Carolina, USA) and SPSS for Mac 21.0 (Chicago, Illinois, USA). A *p* value less than 0.05 was considered statistically significant. The study protocol was approved by the institutional review board.

## Results

### Patients’ characteristics

After applying the inclusion and exclusion criteria, a total of 708 patients were recruited in the analysis. A total of 34 individuals (4.58%) were not included in the study because they started prophylactic anticoagulation during follow-up. Table 1 summarises the main clinical characteristics of these subjects. The majority of patients were female with solid tumours other than those assigned as ‘high’ or ‘very-high’ risk according to the KRS criteria. A total of 30.2% (*n* = 213), 52.9% (*n* = 373) and 16.9% (*n* = 119) of patients were classified as low, intermediate and high risk for VTE according to the KRS.

### VTE cumulative incidence

**Figure 1.**
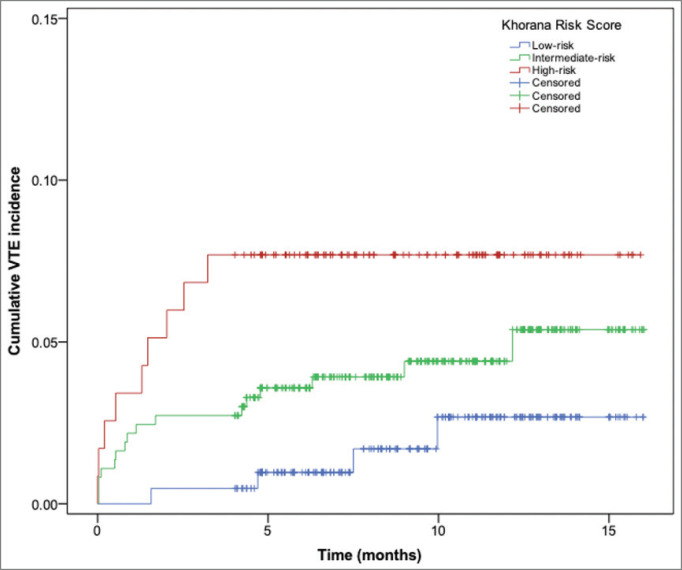
Cumulative VTE incidence (Fine & Gray competing risk model) according to the Khorana risk categories.

Overall, we observed a total of 30 VTE events. The majority of these episodes (*n* = 17) occurred among patients classified as ‘intermediate-risk’, followed by the categories ‘high’ (*n* = 9), and ‘low-risk’ (*n* = 4). The cumulative incidence of VTE at 6 months (considering competing risk of death) was 4.45% (95% confidence interval (CI): 3.25%–6.91%) for the total population. As depicted in Figure 1, the cumulative incidence of VTE at 6 months was 1.56% (95% CI: 0.83%–6.82%), 4.83% (95% CI: 2.81%–7.66%) and 8.84% (95% CI: 4.30%–15.42%) for low-, intermediate- and high-risk Khorana score categories, respectively (Gray’s *p* value: 0.0178).

### Khorana risk score performance

**Figure 2.**
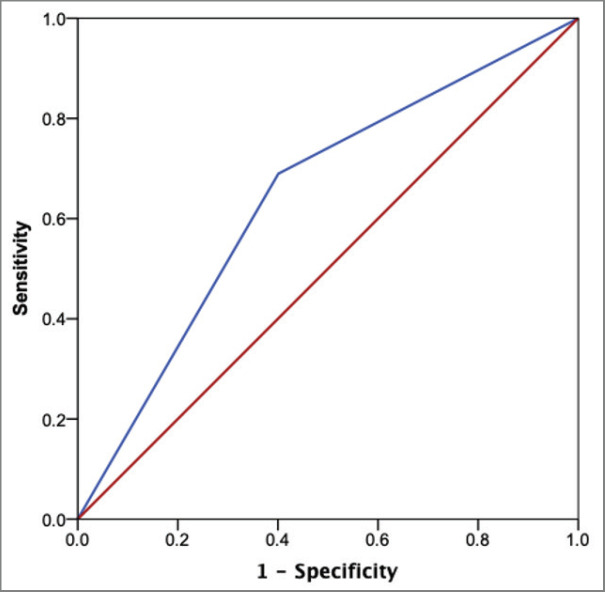
ROC curve using the KRS of 2 as cutoff value.

The ROC curve for the prediction of KRS equal or larger than 2 is presented in Figure 2. This cutoff provided the greatest area under the curve (AUC) (0.65; 95% CI: 0.55–0.75) among the values of 1 (0.58; 95% CI: 0.49–0.68) and 3 (0.57; 95% CI: 0.46–0.68). A KRS cutoff of 2 provided a sensitivity of 69.0% and a specificity of 60% for the prediction of VTE among the included individuals.

### Khorana risk score and overall survival

**Figure 3.**
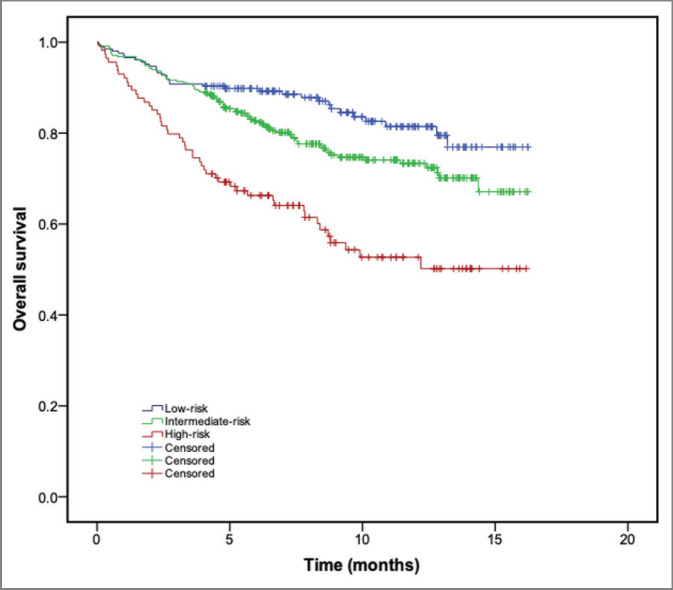
OS (Kaplan–Meier method) according to the Khorana risk categories.

At a median follow-up of 8.13 months (interquartile range: 5.2–11.7 months), a total of 166 patients died (*n* = 24.7%). The diagnosis of any VTE event was associated with an increased risk of death (hazard ratio (HR): 3.02; 95% CI: 1.83–4.98; *p* < 0.001). One-year OS was 81.0%, 73.4% and 50.0% for patients with low-, intermediate- and high-Khorana risk category, respectively. Compared to those patients with low-risk Khorana score, patients classified as ‘high-risk’ had worse OS, both in the univariate (HR: 1.83; 95% CI: 1.46–2.29; *p* < 0.001) and multivariate analysis (HR: 1.62; 95% CI: 1.27–2.05; *p* < 0.001). In the multivariate analysis, advanced age (HR: 1.03; 95% CI: 1.02–1.04; *p* < 0.001) and poor performance status (HR: 2.03; 95% CI: 1.64–2.52; *p* < 0.001) were also associated with worse survival. Figure 3 shows the probability of OS across KRS categories. At the end of follow-up, none of the categories had reached median OS.

## Discussion

In this study, we confirmed the prognostic value of the KRS to predict VTE and mortality in a cohort of ambulatory patients with solid tumours from Costa Rica. To the best of our knowledge, our findings are novel to show the association between the KRS and these clinical outcomes in Hispanic patients from a Latin American country. Therefore, risk stratification through the KRS in our population could improve the identification of patients at risk for VTE, in which the potential benefit of thromboprophylaxis might outweigh the harms of this therapy [17].

Despite current recommendations [6, 7], few patients of this cohort (4.58%) were excluded from analysis because they received prophylactic anticoagulation during follow-up. In agreement with this fact, previous studies have also detected unjustified low rates of thromboprophylaxis and low adherence to current guidelines, which can lead to significant risk of thromboembolic events among predisposal individuals [18]. Although this low adherence to evidence-based recommendations and the subsequent low proportion of patients with prophylactic anticoagulation in this cohort allowed us to detect a more precise estimate of the incidence of VTE according to the KRS strata, these findings should aware medical oncologist to improve their adherence to guidelines and to assess the risk of thrombosis for every patient at the beginning of systemic treatment given the relatively high proportion of subjects who meet criteria for prophylactic anticoagulation.

Our findings also highlight the prevalence of thromboembolic disease as one leading cause of mortality in cancer patients. Of note, the occurrence of VTE was independently associated with an increased mortality, as previous studies have confirmed [19, 20]. The relatively high VTE rates and mortality of patients in higher KRS categories emphasise the unmet clinical needs of such patients. Of particular interest was the significant relationship between the KRS and the risk of death, as previously described by other authors [3]. Although this relationship seems to be expected by many clinicians, this fact emphasises the relevance of the KRS not only to assess the risk of VTE, but also to determine the probability of poor long-term outcomes in affected patients in which these findings could aid in the clinical decision-making process.

Although the KRS has been extensively evaluated as an easy-to-use tool to identify patients at risk for VTE [5], our findings confirmed a poor overall discriminatory performance, as previous studies [21] have revealed, especially in patients with pancreatic [22, 23], lung [24, 25] and gastric cancer [26]. Nevertheless, the majority of cancer patients who developed VTE during follow-up were identified by this score, specifically in the intermediate- and high-risk category. Therefore, it is of value to consider this score in our population given its prognosis impact.

As depicted in Figure 1, the risk of VTE most sharply increased during the first 5 months after initiation of chemotherapy, as previous studies have also described [27]. One strength of this study was the use of a competing risk approach to calculate the cumulative incidence of VTE. In contrast to the Kaplan–Meier method, the Fine & Gray proportional subhazards model is preferred when competing events, such as death, prevent the thrombotic event from being observed [28]. Indeed, previous studies have reported that the Kaplan–Meier method can bias towards an overestimation of cumulative incidences of VTE events [29].

The cumulative incidence of VTE reported in this study was higher than that described for a Caucasian population with a similar method [9]. This difference underlines the relevance of estimating the occurrence of VTE events in different populations since ethnic variation can alter the rate of this outcome. Previous studies have described that cancer patients of Hispanic background are at higher risk of mortality due to pulmonary embolism in comparison with White individuals [12–14]; however, these analyses have also reported that Hispanic patients have lower rates of VTE than White and Caucasian populations. These contradictory results can be explained by several reasons. For instance, these estimations were performed under different methods of analysis and in migrant Hispanic populations with low access to health security systems.

Our study has several limitations by its retrospective nature and the inherent confounders and biases associated with such analysis. Besides, its unicentre design limits the external validity of the findings. Lastly, the adjudication of VTE was retrospective through examination of electronic medical records, but we cannot exclude information bias. Despite these caveats, our study confirms the prognostic value of the KRS to assess the risk of VTE and mortality in a cohort of Hispanic outpatients with solid tumours.

Future studies should compare other risk scores to better estimate the risk of VTE among Hispanic patients. Besides, a calibration of this prognostic tool in this specific population is warranted to improve the accuracy of the KRS according to baseline characteristics, such as ethnicity. In addition, these findings should aware clinicians to stratify cancer patients according to their risk of VTE in order to offer thromboprophylaxis and prevent the occurrence of such events in selected individuals.

## Conclusion

In conclusion, the findings of this ‘real-world’ setting among Hispanic outpatients with cancer add more evidence to the published data describing the predictive role of the KRS for VTE and its impact on OS.

## Conflicts of interest

None to declare.

## Funding

There is no funding to declare.

## Authors’ contributions

AR designed the research, analysed, performed statistical analysis and interpreted data. GH, AM, CU, AC and RM collected and analysed data. All authors wrote the manuscript.

## Figures and Tables

**Table 1.** Clinical characteristics of included patients.

Variable	Value (*n* = 708)
Sex, No. (%) Female Male	443 (62.6) 265 (37.4)
Age, years (mean)	59.04 ± 14.54
ECOG performance status (%) 0 1 2 3	415 (64.8) 181 (28.3) 43 (6.7) 1 (0.2)
Body mass index, Kg/m^2^ (mean)	26.49 ± 6.08
Complete blood counts at diagnosis median (range) White blood cells (cells/µL) Haemoglobin (g/dL) Platelets (cells/µL)	8,100 (2,000–23,100) 12.5 (6.0–19.0) 323,500 (20,000–803,000)
Type of malignancy Breast cancer Colon cancer Gastric cancer Gynaecologic cancer Pancreas cancer Prostate cancer Germ-cell cancer Lung cancer Sarcoma Bladder cancer Other	194 (24.6) 130 (18.4) 97 (13.7) 71 (10.0) 30 (4.2) 30 (4.2) 19 (2.7) 18 (2.5) 12 (1.7) 7 (1.0) 100 (14.1)
